# Accuracy of core needle biopsy for histologic diagnosis of soft tissue sarcoma

**DOI:** 10.1038/s41598-022-05752-4

**Published:** 2022-02-03

**Authors:** J. Kiefer, M. Mutschler, Ph. Kurz, G. B. Stark, H. Bannasch, F. Simunovic

**Affiliations:** 1 Department of Plastic and Hand Surgery, Freiburg University Medical Center, Freiburg, Germany; 2 Department of Pathology, Freiburg University Medical Center, Freiburg, Germany; 3 Department of Plastic, Hand and Aesthetic Surgery, Schwarzwald-Baar-Klinikum Villingen-Schwenningen, Villingen-Schwenningen, Germany

**Keywords:** Surgical oncology, Sarcoma

## Abstract

The biopsy technique of choice in soft tissue sarcoma (STS) diagnosis is controversial. We examined the diagnostic accuracy of percutaneous core needle biopsy (CNB) and compared it to open incisional biopsy. A retrospective study included 91 incisional biopsies and 102 CNBs. A pair-match investigation was conducted on 19 patient pairs, comparing sensitivity, specificity, and diagnostic accuracy. Furthermore, we investigated the role of molecular pathology in sarcoma diagnostics. In 81/91 (89%) patients with incisional biopsy, the entity was confirmed by definitive pathology, whereas this was the case in 89/102 (87%) CNB patients (*p* = 0.52). Grading remained unchanged in 46/55 (84%) of incisional and 54/62 (87%) of CNBs (*p* = 0.61). The pair matched analysis showed that the correct entity was determined in 96% of incisional and 97.6% of core needle biopsies. The time between the initial consultation and the interdisciplinary tumor board's treatment recommendation was shorter in core needle biopsies (8.37 vs. 15.63 days; *p* < 0.002). Incisional biopsies led to two wound infections and one hematoma, whereas wound infection occurred in one patient after CNB. CNB leads to faster diagnosis while reaching the same histological accuracy and is less burdensome for patients. Still, surgeons need to remain aware of the possibility of biopsy failure.

## Introduction

Soft tissue sarcomas are a rare and highly heterogeneous group of malignancies, with over 50 entities^1,2^. Classification of sarcoma is undergoing regular revision, and research into molecular mechanisms of development and progression of sarcoma subtypes continues to translate into pathological diagnostics^3–5^. Correct histological diagnosis and tumor grading are paramount, as they dictate the type and sequence of therapeutic modalities. Traditionally open incisional biopsy delivered the best quality tissue for an accurate diagnosis with the lowest rate of false biopsies. However, this procedure requires general anesthesia, and complications such as hematoma and wound infection occur in up to 16% of cases^6^. Such complications reduce patients' well-being and have adverse effects on the overall treatment outcome, as they can cause tumor spread and delay neoadjuvant treatment.

Less-invasive soft tissue tumor biopsy methods, such as percutaneous core needle biopsy (CNB) and fine-needle aspiration biopsy, have emerged as alternatives. These procedures are performed in the office under local anesthesia. Obvious benefits include faster diagnosis, a lower burden for the patient and the hospital resources, and a lower complication rate. Downsides of percutaneous methods are the inability to visually discern between tumor and normal surrounding tissue, leading to a higher rate of false-negative biopsies, difficulty in accounting for the heterogeneous architecture of specific tumors, technical issues within anatomically challenging locations, and a limited amount of tissue gained.

Accordingly, earlier reports have criticized the lower accuracy of CNB concerning tumor entity and grading^7–12^. A 2009 meta-analysis on 35 studies has found that incisional biopsy has had the highest diagnostic accuracy, followed by CNB, which has been more accurate than fine-needle aspiration biopsy^8^. A single prospective work, which has compared all three biopsy techniques on every tumor, has confirmed the three methods' proposed hierarchy^6^. Newer studies, including a comprehensive 2020 meta-analysis, have reported comparable accuracy of percutaneous CNB and incisional biopsy, and an increasing number of centers have cited CNB as the method of the first choice in diagnosing soft tissue tumors^13–23^. Even though the literature is heterogeneous regarding the most exact biopsy procedure, a trend over the last decade seems to favor CNB. CNB has thus found recognition as the method of choice in the 2018 guidelines of the National Comprehensive Cancer Network (NCCN) for soft tissue tumor biopsy^24^.

This study aims to report our institutional experience and investigate the diagnostic accuracy, sensitivity, specificity, safety, and time to diagnosis of CNB in comparison to open incisional biopsy. Furthermore, we analyze the role of molecular pathology in sarcoma diagnostics in our institution.

## Patients and methods

This study is based on a prospective and continuously updated database of patients with suspected soft tissue sarcoma referred to the Department of Plastic and Hand Surgery at the Freiburg University Medical Center between 2003 and December 2020. The institution is a tertiary referral center and an academic teaching hospital. We introduced the core needle biopsy in our clinic in July 2015. This study reviewed all patients with suspected malignancy, excluding patients with radiologically and clinically inconspicuous masses (e.g., lipoma). Data were compiled from medical records as well as pathology reports and collected anonymously. Patient demographics, tumor type, localization, tumor grading, modality of neoadjuvant and adjuvant therapy and anatomical distribution were collected. The average age at the time of the first operation was 58.95 ± 16.3 in the IB and 61.32 ± 18.48 in the CNB group (*p* = 0.47) years.

National Comprehensive Cancer Network (NCCN) and M stage, grading, anatomical distribution, as well as the type of neoadjuvant and adjuvant radio- and chemotherapy were recorded (Table 1).

**Table 1:** Overview of patients included in the study.

	Incisional biopsy (%)	Core needle biopsy (%)	p- value	Total (% in category)
N	91	102		193
**Gender**				
m	49 (54)	57 (56)	0.88	104 (54)
w	42 (46)	45 (44)		89 (46)
Age (mean ± SD)	58.95 ± 16.3	61.32 ± 18.48	0.47	
**Entity**				
Undifferentiated sarcoma	11 (12)	30 (29)	0.0045	41 (21)
Liposarcoma^π^	27 (30)	14 (14)	0.08	41 (21)
Fibroblastic/myofibroblastic sarcoma^+^	20 (22)	10 (10)	0.028	30 (16)
Vascular sarcoma (mainly angiosarcoma, but also hemangioendothelioma and Kaposi sarcoma)	11 (12)	2 (2)	0.007	13 (7)
Muscle sarcoma (rhabdomyosarcoma and leiomyosarcoma)	5 (5)	2 (2)	0.25	7 (4)
Extraskeletal osteosarcoma and soft-tissue chondroma	6 (7)	9 (9)	0.6	15 (8)
Synovial sarcoma	6 (7)	4 (4)	0.52	10 (5)
Desmoid fibromatosis and myxoma	5 (5)	5 (5)	1.00	10 (5)
Soft tissue metastasis (adenocarcinoma, non-solid tumors)	0	26 (25)	0.0001	26 (13)
**M-status**				
M0	85 (93)	68 (67)	0.001	153 (79)
M1	6 (7)	34 (33)		40 (21)
Grading*				
3	28 (51)	42 (68)	0.03	70 (60)
2	16 (29)	15 (24)		31 (26)
1	11 (20)	5 (8)		16 (14)
**NCCN stage**				
I				
A	3 (4)	2 (5)	0.22	5 (4)
B	17 (22)	7 (18)		24 (21)
II				
A	10 (13)	3 (8)		13 (11)
B	13 (17)	3 (8)		16 (14)
III	28 (37)	15 (39)		43 (38)
IV	5 (7)	8 (21)		13 (11)
**Radiotherapy**				
Neoadjuvant	9 (10)	22 (22)	0.02	31
Intraoperative	12 (13)	11 (11)	0.66	
Adjuvant	32 (35)	11 (11)	0.001	
**Chemotherapy**				
Neoadjuvant	7 (8)	4 (4)	0.3	
Adjuvant	5 (5)	3 (3)	0.47	
**Anatomical distribution**				
Upper extremity	16 (18)	16(15)	0.37	32 (17)
Lower extremity	59 (65)	57 (56)		116 (60)
Trunk	11 (12)	20 (20)		31 (16)
Head and neck	8 (5)	9 (9)		14 (7)

^π^Includes tumors classified as “round cell” before the 2013 Classification; ^+^includes dermatofibrosarcoma protuberans, malignant solitary fibrous tumor, malignant fibrous histiocytoma (entity removed from the classification in the 2013 WHO Classification of Tumors of Soft Tissue); *on biopsy, not attempted on all specimens, not reported for specimens that have undergone neo-adjuvant irradiation, and not reported for non-sarcoma tumors.

**Figure 1:**
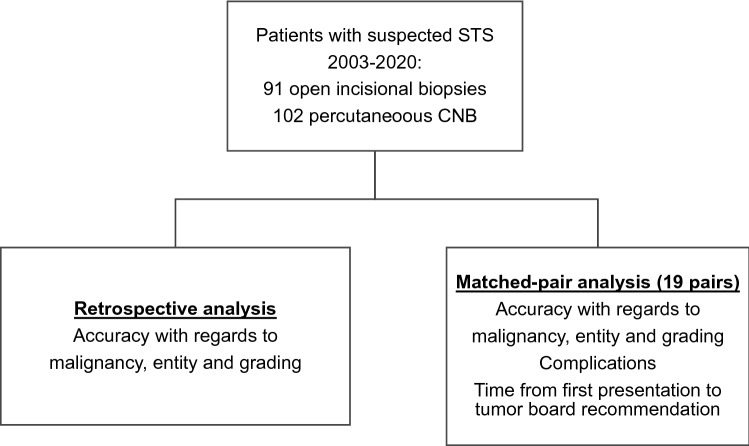
Patients with suspected soft tissue sarcoma (SFT) between 2003 and 2019 were included in the study. The core needle biopsy (CNB) was introduced in our department in July 2015, and since then, 102 patients have been diagnosed with CNB. These were compared to 91 open incisional biopsies regarding accuracy in diagnosing the correct entity and tumor grading. Secondly, a pair-matched analysis was conducted on 19 pairs, matching gender, age, and tumor characteristics: histology, localization, size, depth, and grading. The pairs were compared for sensitivity, specificity, the accuracy of diagnosis, complications, and time to case presentation in the interdisciplinary tumor board.

In the first part of the study, we retrospectively reviewed all biopsies, recording the number of samples, the modality of molecular diagnostics performed, tumor entity, and tumor grading (Fig. 1). We then compared these findings to the definitive resection specimens to evaluate the accuracy of diagnosis regarding malignancy, tumor entity, and grading. In the second part of the study, we performed a matched-pair analysis according to the following criteria: gender, age, tumor entity, localization, size and depth of the tumor (epi-/subfascial), and grading (Fig. 1). We assigned 38 patients to 19 pairs, which we further analyzed regarding diagnostic accuracy and time from initial consultation to case presentation in the interdisciplinary tumor board, time from initial consultation to initiation of therapy, and time from biopsy to initiation of therapy. Complications, such as wound infection and hematoma, were noted. No patients were lost to follow up, as they all proceeded to receive treatment at the Freiburg University Medical Center. We further reviewed the diagnostic accuracy as a function of time, asking whether there was a change in diagnostic accuracy during the study period. Finally, we reviewed whether the use of molecular testing was associated with higher diagnostic accuracy and which gene alterations and mutations were most frequently found in our population.

**Figure 2:**
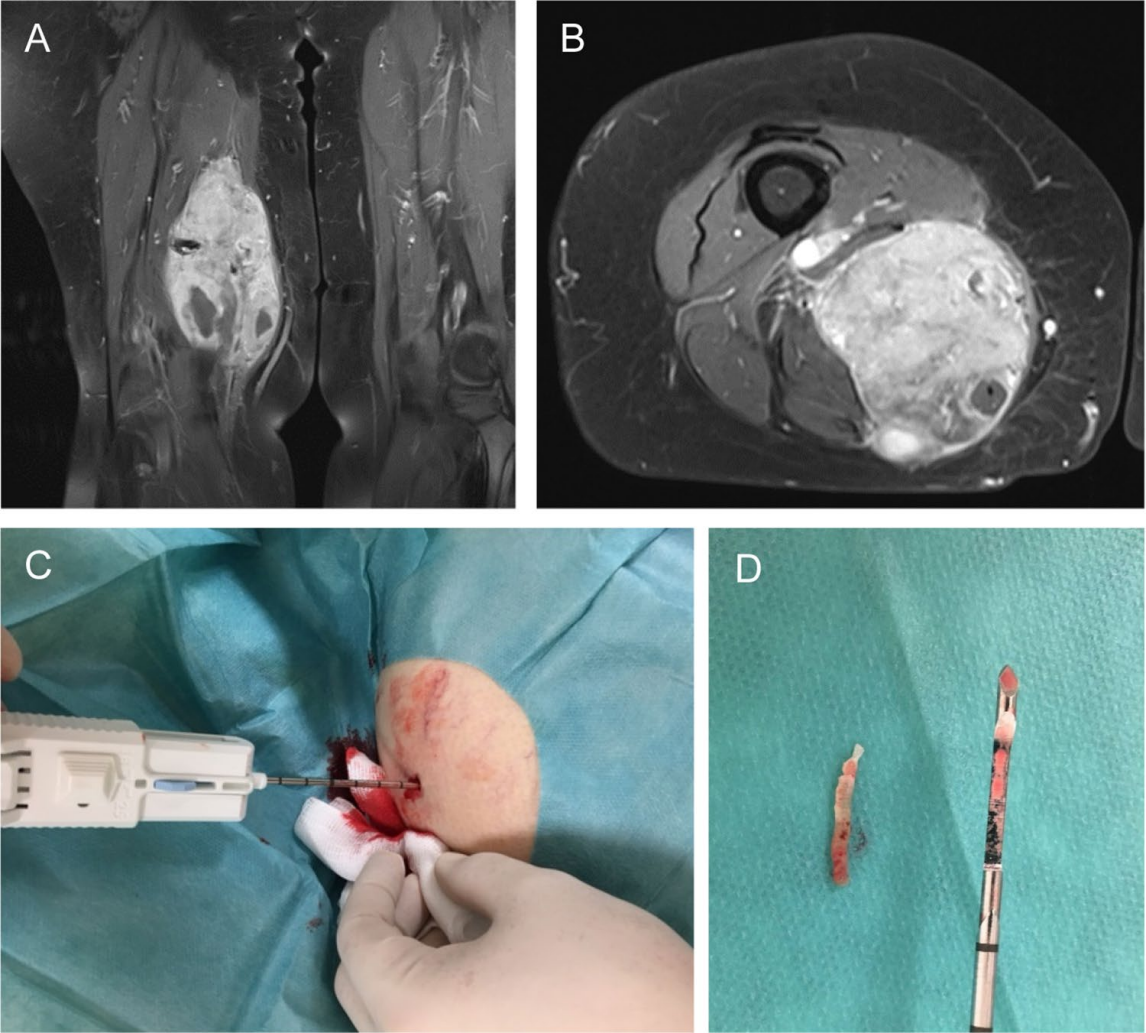
A 78-year old patient was referred to us with a large, palpable mass in the left leg. An MRI showed a 15 × 9 cm heterogeneous, contrast-enhanced tumor (**A**,**B**), which was biopsied using a HistoCore core needle biopsy device (**C**,**D**). One day after the initial consultation, the diagnosis of a high-grade sarcoma was made by the pathologist. Additional immunohistochemistry specified the diagnosis of an undifferentiated pleomorphic sarcoma five days following biopsy, enabling the interdisciplinary discussion in the tumor board seven days after the initial consultation.

A board-certified plastic surgeon specializing in sarcoma surgery performed all biopsies, tumor resection, and reconstruction. The CNBs were performed in an outpatient setting, usually during the initial consultation. After obtaining informed consent, local anesthesia was administered, and the site of the biopsy was prepared. The surgeon then carefully examined the preoperative MRI scans for localization and heterogeneity of the soft tissue tumor to determine which section of the mass was most representative for biopsy. The skin was incised with an 11° blade and the biopsy was performed using the 12 Gauge BIP-HistoCore HC needle (Biomed. Instrumente & Produkte GmbH, Türkenheim, Germany). If possible, the mass was palpated by the non-dominant hand during the biopsy (Fig. 2). In some cases, sonographic guidance was used to optimize the accuracy of the CNB. At least three tissue probes (mean 3.59 ± 2; min. 1 and max. 12) were taken and examined visually, as the tumor tissue could often be differentiated macroscopically from fatty or muscle tissue. Tissue cylinders were then sent for pathologic analysis in formaldehyde.

Incisional biopsies were performed under general anesthesia on an in-patient basis. Following the skin incision, the soft tissue compartments were dissected until the tumor was identified, and a representative section of the tumor mass was biopsied under visual control through a wedge resection. Following pathology workup and staging, the case was presented at the interdisciplinary sarcoma board of the Freiburg University Medical Center, where the type and sequence of therapeutic modalities were discussed. Neoadjuvant radiotherapy is recommended for all high-grade (G2 and G3) tumors and was accompanied by intraoperative radiation when the STS was localized close to vital anatomic structures that might preclude achieving sufficient resection margins. Patients with chemosensitive tumors (rhabdomyosarcoma, synovial sarcoma, and extraosseous Ewing’s sarcoma) were advised to receive neoadjuvant and adjuvant chemotherapy. Our approach to treatment and surgical strategy was extensively published previously^25–33^. Union for International Cancer Control (UICC) 7th edition is used for staging, and Fédération Nationale des Centres de Lutte Contre Le Cancer (FNCLCC) classification is used for grading tumors.

To compare the groups and to analyze test accuracy, four-field tables were created. The groups were compared using Fisher's exact test. Wilcoxon signed-rank test was used to compare the time from biopsy to receiving a tumor board decision. The analysis was performed using Stata 14 (StataCorp LP, 4905 Lakeway Drive, College Station, TX 77845, USA), and results were considered significant if *p* < 0.05. The Ethics committee of the Faculty of Medicine, University of Freiburg approved this study (282/19), and the need for informed consent was waived. All methods were carried out in accordance with relevant guidelines and regulations. The datasets are available from the corresponding author on request.

## Results

Of 193 cancer patients included in the study, 91 had incisional biopsies and 102 CNB. 89/193 (46%) of patients were female. The most common sarcoma subtype in our population was undifferentiated sarcoma (21%), followed by liposarcoma (21%), fibroblastic or myofibroblastic sarcomas (16%), and liposarcoma (17%) (excluding atypical lipomatous tumors, G1 liposarcoma). In the CNB group, we biopsied significantly more undifferentiated sarcomas and soft tissue metastases of other adenocarcinoma or non-solid tumors, whereas we found more sarcomas arising from vascular tissue in the incisional biopsy group. At the initial presentation, 79% of patients had no distant metastasis. The CNB population presented with a significantly higher rate of metastases. 60% of patients had a G3 STS, 26% a G2, and 14% a G1 tumor, with CNB patients exhibiting significantly less differentiated (higher G) tumors. The distribution of NCCN stages, administration of chemotherapy, and anatomical distribution were even across both groups. CNB patients underwent neoadjuvant radiation therapy in more cases than patients diagnosed by incisional biopsy (Table 1).

Analysis of all patients regarding the accuracy of biopsy techniques revealed a 96% accuracy for diagnosing malignancy via CNB and 100% for incisional biopsy. Tumor subtype diagnosis was confirmed by pathology on the definitive specimen in 89/102 (87%) CNB cases and 81/91 (89%) incisional biopsy cases (*p* = 0.52). Grading was correct in 54 (87%) CNB specimens and 46 (84%) incisional biopsy specimens (*p* = 0.61). When excluding non-sarcoma tumors from the analysis the accuracy of tumor subtype diagnosis was at 83% for CNB and 89% for IB (*p* = 0.25) and the accuracy of grading at 88% and 84%, respectively (*p* = 0.59, Table 2).

**Table 2:** Analysis of all patients (N = 193) reporting the accuracy of incisional and core needle biopsies with regards to diagnosis of malignancy and accuracy in diagnosing entity and grading.

	Biopsy result		*P*-value
	CNB (%) N = 102	IB (%) N = 91	
Malignancy	98 (96)	92 (100)	0.91
Tumor subtype	89 (87)	81 (89)	0.52
Grading	81 (91)	46 (84)	0.19
**Accuracy of biopsy excluding metastases and other non-sarcoma tumors**			
Tumor subtype	63 (83)	81 (89)	0.25
Grading	53 (88)	46 (84)	0.59

Accuracy is defined as agreement between pathology results on biopsy tissue and on the surgical resection specimen. Grading was diagnosed in 89 CNB and 55 incisional biopsy cases. Since the CNB group included 26 cases of non-sarcoma tumors, the accuracy of biopsies was separately calculated excluding these tumors.

*CNB* core needle biopsy; *IB* incisional biopsy.

For the matched-pair analysis, we created 19 patient pairs that matched according to gender, age, and tumor characteristics: entity, localization, size, depth, and grading. Matching of gender and tumor grading was possible in all pairs. A match in at least 5 of 7 tumor criteria was required to build a pair. Supplementary Table 1 gives an overview of the pairs. Sixteen of 19 cases had a G3, > T3 sarcoma of the lower extremity. The correct entity was diagnosed in nearly all cases, and the correct grading was diagnosed in 90% of incisional biopsies and 94% of CNBs (*p* = 0.62). The time from the initial consultation until case presentation and discussion in the interdisciplinary tumor board was significantly shorter for the CNB group (8.4 ± 5 days) compared to the incisional biopsy group (15.6 ± 6.6 days, *p* < 0.002). Time from initial consultation to initiation of therapy (surgical or neoadjuvant) was also shorter, however not significantly (22.83 ± 17.4 vs. 28.83 ± 18.6 days, *p* = 0.32). Time from biopsy to initiation of therapy was not different (22.44 ± 17.5 vs. 24.11 ± 17.5, *p* = 0.77). Amongst 19 incisional biopsies, two wound infections and one hematoma requiring revision occurred. There was one wound infection after CNB (Table 3).

**Table 3:** Results of the pair-matched analysis on 19 pairs matched according to gender, age, and tumor characteristics: entity, localization, size, depth, and grading.

	Incisional biopsy	Core needle biopsy	p
Sensitivity (malignancy)	100%	100%	
Positive predictive value (malignancy)	100%	100%	
Correct entity	96%	98%	n.s
Correct grading	90%	94%	0.62
Time from initial consultation to case presentation in tumor board (days)	15.6 ± 6.6	8.4 ± 5	< 0.002
Time from initial consultation to initiation of therapy* (days)	28.83 ± 18.6	22.83 ± 17.4	0.32
Time from biopsy to initiation of therapy* (days)	24.11 ± 17.5	22.44 ± 17.5	0.77
Time from biopsy to case presentation in tumor board (days)	15.6 ± 6.6	8.4 ± 5	< 0.002
Anesthesia	15 general	19 local	
Complications	2 wound infections 1 hematoma requiring revision	1 wound infection	

Sixteen of 19 cases had a G3, > T3 sarcoma of the lower extremity.

*Surgery, or beginning of neoadjuvant chemotherapy or radiation therapy.

**Figure 3:**
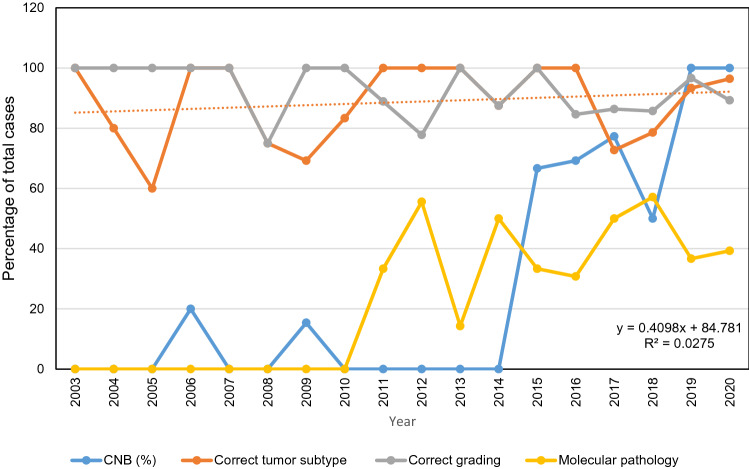
The level of diagnostic accuracy with regards to tumor type increased during the study period (R^2^ = 0.02). Results for tumor accuracy have been fitted to create the regression line. An increase in molecular pathological testing is noted beginning in 2011, and an increase in the use of core needle biopsy (CNB) is noted from 2015.

During the study period, the level of diagnostic accuracy regarding tumor subtype increased (R^2^ = 0.02). Molecular diagnostics for sarcoma were introduced in our institution in 2011, and CNB in 2015 (Fig. 3). Interestingly, the use of molecular diagnostics did not increase diagnostic accuracy with regards to tumor subtype. Out of 61 cases in which molecular pathology was performed, the correct diagnosis was achieved in 54 (88%) of cases. The same percentage of diagnostic accuracy was seen in patients in which molecular pathology was not used (116 out of 132, 88%, *p* = 1). The most common molecular tests performed in this series are listed in Table 4. It is of clinical significance to ask whether the diagnostic discrepancy between CNB and the definitive specimen pathology influenced patient treatment. Table 5 lists these discrepancies. In one case, a more accurate tumor diagnosis might have resulted in recommending a different neoadjuvant therapy. We found most discrepancies in grading regarding the difference between G2 and G3 STS (6 cases). For most sarcoma entities, both G2 and G3 graded tumors underwent neoadjuvant radiation, rendering this discrepancy clinically inconsequential. Two cases had a discrepancy between G1 and G2, which prevented the recommendation for neoadjuvant radiation therapy in these cases.

**Table 4:** Overview of genes targeted by molecular diagnostics in this study.

Gene product	Sarcoma subtype	Affected pathway	Incisional biopsy	Core needle biopsy	Total
MDM2	Liposarcoma	Lack of function of the p53 tumor suppressor	12	3	15
CDK4	Liposarcoma	Cyclin-dependent kinase	14	3	17
DDIT3	Myxoid/round cell liposarcoma	CCAAT/enhancer-binding protein family (pro-apoptotic transcription factors)	12	7	19
SS18	Synovial sarcoma	SWI/SNF chromatin remodeling complex	7	5	12
EWSR family members	Ewing sarcoma	FET family of transcriptional regulators	8	1	9
GNAS	Intramuscular myxoma	Activation of cAMP-Dependent protein kinase A	1	0	1
CTNNB1	Desmoid tumors	Beta-catenin (regulation of cell–cell adhesion and gene transcription)	1	2	3
RNA fusion pannel			6	0	6
Others*			8	2	10

*EBV, BCL family members, Myc, USP6.

**Table 5:** In 13 patients, the final diagnosis differed from the core needle biopsy (CNB) diagnosis.

Patient	Age	Localization	CNB diagnosis	Final diagnosis	Influence on treatment
1	58	Shoulder	Myxoid liposarcoma	Myxoid chondro-sarcoma	Yes, neoadjuvant chemotherapy would likely have been proposed for chondro-sarcoma
2	70	Abdomen	Pleomorphic sarcoma	Inflammatory fibrocystic histiocytoma	No
3	85	Thigh	Myxoid sarcoma	Pleomorphic sarcoma	No
4	78	Forearm	Myxofibrosar-coma	Pleomorphic spindle cell sarcoma	No

In four patients, CNB failed to diagnose malignancy. In two patients, diagnosis of the malignant tumor was achieved, but not the subtype. In four cases, subtype diagnosis was attempted but differed from the definitive diagnosis. The latter four cases are listed here, reflecting whether the incorrect initial diagnosis influenced the treatment. This seems to have been the case in only one patient.

### Case presentations

We chose two clinical cases to illustrate the advantages and disadvantages of CNB. Figure 2 demonstrates the most common scenario of a tumor that is easily accessible to CNB. A 78-year old patient presented with a large, palpable mass in the left thigh. MRI showed a 15 × 9 cm heterogeneous, contrast-enhanced tumor (A and B), which we biopsied using a HistoCore core needle biopsy device (C and D). One day after the initial consultation, the histological workup revealed the diagnosis of high-grade sarcoma. Additional immunohistochemistry specified the diagnosis of an undifferentiated pleomorphic sarcoma five days following biopsy, enabling the interdisciplinary discussion in the tumor board seven days after the initial consultation.

**Figure 4:**
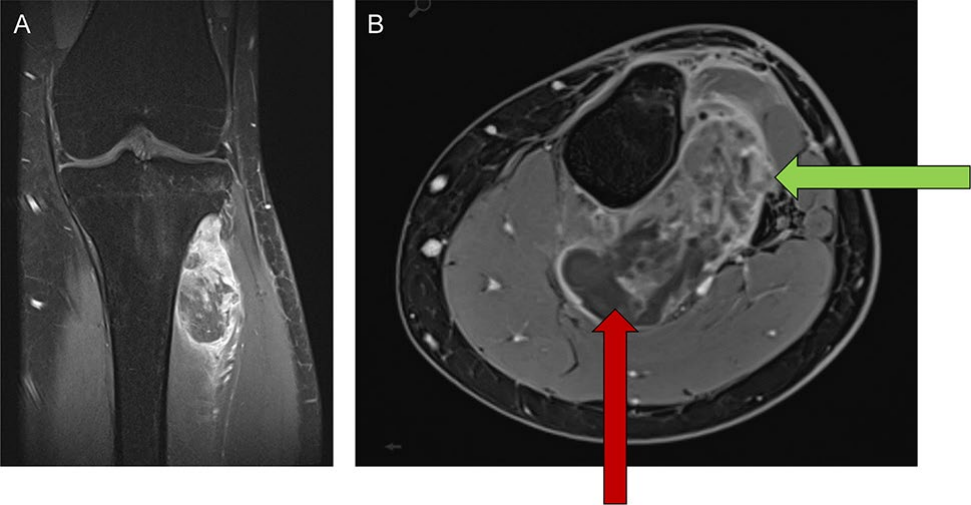
Coronal and axial magnetic resonance images showing a heterogeneous contrast-enhanced tumor of the leg in a 19-year-old girl. Two percutaneous CNB from the posterior region of the tumor were performed (red arrow in **B**) because the ventral part of the tumor had a close relationship to the peroneal nerve. Both biopsies resulted in inconclusive pathology. An open biopsy was required from a more contrast-enhancing ventral region (green arrow in **B**) to diagnose extraskeletal Ewing's sarcoma. The estimated delay to diagnosis from the two failed CNBs, in this case, was two and a half weeks. This case illustrates the importance of persistence in acquiring the diagnosis as a negative CNB result does not exclude malignancy. The ability to correlate clinical, radiological, and pathological findings is crucial.

Figure 4 shows coronal and axial magnetic resonance images of a heterogeneous contrast-enhanced tumor of the lower leg in a 19-year-old girl. Here, two percutaneous CNBs from the posterior region of the tumor were attempted (red arrow in B) because the ventral part of the tumor had a close relationship to the peroneal nerve. Both biopsies resulted in inconclusive pathology. Thus, we performed an open biopsy from a more contrast-enhancing ventral region (green arrow in B) to diagnose extraskeletal Ewing's sarcoma. The estimated delay to diagnosis from the two failed CNBs, in this case, was two and a half weeks. In this case, persistence in acquiring the diagnosis was essential, as a negative CNB result does not exclude malignancy.

Apart from this case, CNB failed to diagnose malignancy in three other cases in this series. In these cases, imaging was strongly suggestive of undifferentiated sarcoma, and biopsy showed necrotic tissue, also strongly suggestive of malignancy. Therefore there was no need for new biopsy and primary surgery has been suggested, so that there was no delay in treatment.

## Discussion

This retrospective analysis provides evidence that core needle biopsy is as accurate as incisional biopsy in diagnosing soft tissue sarcoma. CNB achieved histological accuracy for subtype diagnosis in 87% of cases compared to 89% from incisional biopsy. As confirmed by pathology on the definitive specimen, tumor grading was correct in 87% of CNB specimens compared to 84% of incisional biopsy specimens. In addition, CNB led to faster diagnosis of malignancy while being less burdensome for patients and clinical resources. Performing CNB led to a shorter time between the initial presentation and tumor board decision than performing IB. Time from the initial presentation and initiation of therapy was also shorter in the CNB group, but not significantly.

During the last decade, reports have been accumulating on the reliability of CNB for the primary diagnosis of soft tissue tumors^13–22^. A recent meta-analysis including 17 studies and 2680 patients confirmed that CNB has high accuracy in diagnosing the lesions with fewer complications than incisional biopsy^23^. Thus, in many centers, including ours, CNB is the primary method for biopsy soft tissue tumors. While the ability to diagnose malignancy by CNB is widely accepted, several authors have reported varying accuracy levels in diagnosing the correct tumor entity and grading^7–12^. Concerning the competing method of fine-needle aspiration biopsy, there is widespread consensus, with very few exceptions^34^, that it is significantly less accurate and, thus, inferior to both open incisional biopsy and CNB^35,36^.

As the matched-pair analysis demonstrated, the most typical case encountered in a soft tissue tumor center is a patient with a large, palpable mass of the thigh and an MRI suggestive of malignancy (Fig. 2). These tumors are easily accessible by CNB, and the matched-pair analysis showed a 100% sensitivity for malignancy and a > 95% correct diagnosis of the entity in both biopsy techniques. With almost 90% correct entities in the analysis of all patients, our study exhibits a relatively high degree of diagnostic accuracy based on either type of biopsy material. The diagnostic accuracy increased during the study period. In the literature, accuracy regarding the histological tumor type has been reported as high as 83%^16^, 80%^13^ or even as low as 45%^6^, and, in two recent large meta-analyses, at 84%^37^ and 88%^23^. Diagnostic accuracy in sarcoma biopsy is multifactorial. We consider the existence of dedicated specialists from every department involved, who stay in close communication with each other, to be of utmost importance for accurate and efficient sarcoma diagnosis. The radiologist and the surgeon need to have sufficient experience in sarcoma diagnosis and treatment to precisely determine which portion of the tumor is most likely to yield significant histologic information. Execution of the biopsy involves precision and critical judgment. Possibly, soft tissue tumors are even better sampled by CNB than incisional biopsy, since CNB allows for fanning out and sampling deeper parts of the mass. We require at least three passes of the needle per tumor (the mean was 3.59 ± 2 in this study), and this amount of tissue is sufficient for all pathological tests^38^. We thus consider the increasing expertise of the interdisciplinary team to be the main factor for the increase in accuracy of the histological diagnosis seen in our analysis. CNB can be performed under ultrasound guidance if needed, but we do not consider computer tomography (CT) or MRI guidance necessary, despite several positive series in the literature^39–41^.

Molecular diagnostics are the most exciting recent development in the sarcoma field. Increased understanding of molecular mechanisms of sarcoma allows us to reconsider the classification system, implying potential clinical consequences^4^. The most commonly investigated gene products were MDM2 and CDK4^42,43^, the mutations of which are markers for liposarcoma. This is followed by DDIT3, mutated in myxoid sarcoma, SS18 (synovial sarcoma^44^), EWSR family members (Ewing sarcoma^45^), and GNAS and CTNNB1 that are mutated in intramuscular myxoma and desmoid fibromatosis^46^, respectively (Table 4).

A prospective multicenter study on 395 patients has demonstrated that molecular diagnostics can change diagnosis in specific STS subtypes^43,47,48^. The authors have cited several tumors that were reclassified as dermatofibrosarcoma protuberans, requiring surgery to reach R0 status, and several cases that were reclassified from the initial diagnosis of Ewing sarcoma, thus sparing these patients from useless chemotherapy. In our series, there were no cases where a change in therapy was affected by molecular pathology findings. For example, the most common molecular tests for MDM2 and CDK4 mutations were used to dedifferentiated liposarcoma and undifferentiated pleomorphic sarcoma, and both entities are treated identically. However, there are suggestions that MDM2 negative undifferentiated pleomorphic sarcomas have a less favorable outcome than MDM2 positive tumors^42,43^. Molecular diagnostics were instrumental in diagnosing desmoid fibromatosis and intramuscular myxoma, as these tumor entities are notoriously challenging to diagnose, but the exact diagnosis does not change the course of therapy. In certain situations, such as differentiation between melanoma and peripheral nerve sheet tumors, molecular diagnostics are essential. After correlating the use of molecular diagnostics and diagnostic accuracy, we observed that molecular diagnostic methods were not associated with higher diagnostic accuracy in this study. Still, we agree that molecular diagnostics should be mandatory for sarcoma diagnosis. In our institution, in-situ hybridization is completed within 1–2 weeks. Thus, there is no delay in therapy. Fusion panels can take up to 4–6 weeks, and they are mostly needed to differentiate non-malignant tumors and low-grade sarcomas.

Future developments in molecular diagnostics will open new avenues of diagnostic and therapeutic possibilities. For instance, we^49–51^ and others^52,53^ explore recurrent sarcoma translocations as targets for “liquid biopsy.” Here, circulating DNA harboring a specific translocation is measured to monitor tumor activity, which has relevance for patient follow-up and assessing the success of multimodal sarcoma therapy. Further developments are expected in the area of targeted molecular therapeutics, which will benefit patients with advanced sarcoma^54^.

In our study, the discrepancy in diagnosis and grading based on definitive resection specimen was, in most cases, not relevant for therapy. Still, there were some cases in which therapy would have been different. For instance, some patients with high-grade sarcomas might benefit from neoadjuvant irradiation, which is not recommended if the biopsy suggests a lower tumor grading (Table 5). It is crucial to discuss the few cases in which CNB failed to diagnose malignancy, requiring incisional biopsy (Fig. 4). A negative CNB does not ensure the absence of malignancy. The ability to correlate clinical, radiological, and pathological findings is crucial, and the clinicians have to persist in achieving the correct diagnosis. Furthermore, it is paramount to place the biopsy in the area of future resection to avoid contaminating healthy tissues with tumor cells and impede tumor resection. For all these reasons, tumor biopsy is not delegable, and the surgeon who will perform the tumor resection needs to perform the biopsy.

Our study included non-malignant tumors relevant to differential diagnosis, such as desmoid tumors and intramuscular myxoma, but excluded lipomas. Looking into differentiation by biopsy between lipomas and atypical lipomatous tumors is an interesting question but one we decided not to investigate here because it does not fit the scope of the present work. At the beginning of the study period, in 2013, a change in sarcoma classification took place^3^. We thus had to classify previously used entities, such as malignant fibrous histiocytoma, in broader categories (see explanation to Table 1). The changes in sarcoma classification did not influence the statistical analysis of the test sensitivity in this study, as each biopsy was compared to the resection specimen, which was diagnosed according to the same classification. The CNB group in this work harbors more advanced and more aggressive tumors (higher rate of metastases and higher grading). This is due to the temporal development in our institution since, with time, our regional reputation as sarcoma center grew, resulting in more advanced cases presenting to us. This difference also has no significance for the interpretation of the current research question.

In light of our data and the pertinent literature, we conclude that CNB is reliable and safe as a method of the first choice for soft tissue sarcoma biopsy. It has the same diagnostic accuracy as incisional biopsy, and the speed and convenience of the method greatly exceeded those of incisional biopsy. However, both its reliability and safety depend on the expertise and high degree of specialization of the surgeon and the pathologist.

## Supplementary Information

Supplementary Information.
