# Supplementary material for: Accuracy of core needle biopsy for histologic diagnosis of soft tissue sarcoma
Source: Sci Rep. 2022 Feb 3;12:1886. doi: 10.1038/s41598-022-05752-4 (PMC8813997; doi:10.1038/s41598-022-05752-4)
Supplement: Supplementary file 1 — Supplementary Information. [file 41598_2022_5752_MOESM1_ESM.docx]

**Accuracy of Core Needle Biopsy for Histologic Diagnosis of Soft Tissue Sarcoma**

J. Kiefer^1^, M. Mutschler^1^, G.B. Stark^1^, H. Bannasch^2^, F. Simunovic^1^

^1^Department of Plastic and Hand Surgery, Freiburg University Medical Center, Freiburg, Germany

^2^Department of Plastic, Hand and Aesthetic Surgery, Schwarzwald-Baar-Klinikum Donaueschingen, Germany

**Supplementary table 1.** Nineteen pairs, which were included in the pair-matched analysis, matched in at least 5 of 7 parameters**.** The conflicting parameter are highlighted in the table. Age was considered matching if patients’ age at time of surgery was ≤5 years apart.

| **Pair** | **Gender** | **Age** | **Entity** | **Localisation** | **Size (cm)** | **Depth (epi-/subfascial)** | **Grading** |
| --- | --- | --- | --- | --- | --- | --- | --- |
| 1 | F | 45/67 | Liposarcoma | Trunk/lower extremity | >5 | Deep | G3 |
| 2 | M | 83/58 | Rhabdomyosarcoma/synovial sarcoma | Head and neck | <5 | Superficial | G3 |
| 3 | M | 81/80 | Pleomorphic sarcoma | Lower extremity | >5 | Deep | G3 |
| 4 | M | 67/77 | Liposarcoma/fibrosarcoma | Lower extremity | >5 | Deep | G3 |
| 5 | M | 59/19 | Myxofibrosarcoma | Lower extremity | >5 | Deep | G2 |
| 6 | M | 90/52 | Leiomyosarcoma/pleomorphic sarcoma | Lower extremity | >5 | Deep | G3 |
| 7 | F | 59/55 | Liposarcoma/fibrosarcoma | Lower extremity | >5 | Deep | G1 |
| 8 | M | 79/77 | Pleomorphic sarcoma/liposarcoma | Lower extremity | >5 | Deep | G3 |
| 9 | F | 64/39 | Pleomorphic sarcoma | Upper/lower extremity | >5 | Deep | G1 |
| 10 | M | 56/63 | Pleomorphic sarcoma | Lower extremity | >5 | Deep | G3 |
| 11 | M | 70/70 | Extraskelletal chondrosarcoma/synovial sarcoma | Upper/lower extremity | >5 | Deep | G3 |
| 12 | M | 80/76 | Pleomorphic sarcoma | Head/lower extremity | <5cm/>5cm | Deep | G3 |
| 13 | M | 80/48 | Angiosarcoma/extraskelletal chondrosarcoma | Upper extremity | <5cm | Deep | G3 |
| 14 | M | 30/52 | Fibrosarcoma/malignant gastrointestinal stromal tumor | Lower extremity | >5 | Deep | G3 |
| 15 | F | 44/74 | Pleomorphic sarcoma | Lower extremity | >5 | Deep/superficial | G3 |
| 16 | F | 35/56 | Fibrosarcoma | Lower extremity | >5 | Deep | G3 |
| 17 | M | 60/49 | Pleomorphic sarcoma | Lower extremity | >5 | Superficial/deep | G3 |
| 18 | F | 78/75 | Pleomorphic sarcoma | Lower extremity | >5 | Deep | G3 |
| 19 | F | 54/59 | Pleomorphic sarcoma/liposarcoma | Lower extremity | >5 | Deep/superficial | G3 |
